# Cardiac surgery during wartime in Israel

**DOI:** 10.1186/s13019-024-02907-4

**Published:** 2024-07-15

**Authors:** Eitan Keizman, Tamer Jamal, Irena Sarantsev, Eilon Ram, Aryel Furman, Alexander Kogan, Ehud Raanani, Leonid Sternik

**Affiliations:** 1 Department of Cardiac Surgery, The Leviev Cardiothoracic and Vascular Center, Sheba Medical Center, Tel Hashomer, Israel; 2 Sackler School of Medicine, Tel Aviv University, Ramat-Gan, Israel; 3 The Edmond J. Safra International Congenital Heart Center, Sheba Medical Center, Tel Hashomer, Israel

**Keywords:** Cardiac surgery, Heart transplantation

## Abstract

**Background:**

The war that began on October 7th, 2023, has impacted all major tertiary medical centers in Israel. In the largest cardiac surgery department in Israel there has been a surprising increase in the number of open-heart procedures, despite having approximately 50% of surgeons recruited to military service. The purpose of this study is to characterize this increase in the number of operations performed during wartime and assess whether the national crisis has affected patient outcomes.

**Methods:**

The study was based on a prospectively collected registry of 275 patients who underwent cardiac surgery or extracorporeal membrane oxygenation (ECMO) during the first two months of war, October 7th 2023 – December 7th 2023, as well as patients that underwent cardiac surgery during the same period of time in 2022 (October 7th, 2022 – December 7th, 2022).

**Results:**

120 patients (43.6%) were operated on in 2022, and 155 (56.4%) during wartime in 2023. This signifies a 33.0% increase in open-heart procedures (109 in 2022 vs. 145 in 2023, p-value 0.26). There were no significant differences in the baseline characteristics of patients when comparing the 2022 patients to those in 2023. No significant differences between the two groups were found with regards to intraoperative characteristics or the type of surgery. However, compared to 2022, there was a 233% increase in the number of transplantations in the 2023 cohort (p-value 0.24). Patient outcomes during wartime were similar to those of 2022, including postoperative complications, length of stay, and mortality.

**Conclusions:**

Patients who underwent cardiac surgery during wartime presented with comparable outcomes when compared to those of last year despite the increase in cardiac surgery workload. There was an increase in the number of transplants this year, attributed to the unfortunate increase in organ donors.

## Introduction

On October 7th 2023, Hamas launched an armed invasion of Israel killing over 1,300 and kidnapping more than 240 innocent citizens. The attack provoked a war in the region that started then and continues through today (January 2024). This war has significantly affected health care facilities in Israel. All major tertiary centers, such as ours, allocated most of their resources towards treating the hundreds of wounded soldiers and civilians. Elective daily activities were reduced and, in addition, hundreds of physicians and healthcare providers were recruited into the Israeli Defense Forces (IDF). As the war began, we anticipated a decrease in daily activity at the cardiac surgery department. Surprisingly, however, we have witnessed a major increase in the number of the non-war related open-heart procedures. This increase in the number of operations during the first two months after October 7th, raises two critical questions regarding cardiac surgery during this wartime period: Do the characteristics of the patients in 2023 differ to those of the same period last year, and does the increased burden along with the lack of personnel affect patient outcomes? The aim of this study is to provide answers to these two questions.

## Methods

### Study design, ethics, and patients

This single-center retrospective observational study of prospectively collected data was performed in the Department of Cardiac Surgery, The Leviev Cardiothoracic and Vascular Center, Sheba Medical Center, Tel Hashomer, Israel. The study was approved by the institutional review board of the Chaim Sheba Medical Center, Tel Hashomer (Approval number: 4527. Date of approval: November 7th, 2023. Patient consent to participate; not relevant). As the number of procedures has been steady in recent years, we have decided to conduct a comparison between patients who underwent cardiac surgery in the first two months of the war to those during the same period of the year before. All patients who had undergone cardiac surgery or an extracorporeal membrane oxygenation (ECMO) procedure from October 7th, 2023 – December 7th 2023 as well as October 7th, 2022 – December 7th, 2022 were included in the study. Patients who underwent minor interventions such as removal of sternal wires, ECMO decannulation, and revision for bleeding were excluded from the cohort. Veno-arterial (VA) ECMO was implemented for patients with cardiogenic shock due to ischemic or non-ischemic cardiomyopathy. Veno-venous (VV) ECMO was utilized for patients with respiratory failure. None of the patients in the cohort required post-cardiotomy VA ECMO. A total of 275 patients were included in the study. 120 patients (43.6%) were operated in 2022 (2022 group) and 155 (56.4%) during wartime in 2023 (2023 group). The patients were compared on the basis of their baseline characteristics, operative reports, and postoperative course.

### Personnel changes

As the war began, medical personnel were reduced due to IDF recruitment. This included a 37.5% decrease in the number of attending surgeons (from 8 to 5) and a 62.5% decrease in the number of residents (from 8 to 3). The transplantation service at our center was operating with 50% less transplant surgeons when compared to the previous year. Nursing staff at the cardiac surgery intensive care unit (CSICU) had also seen a decrease in the number of members, which was, however, less significant. Approximately 10% of the nursing positions were missing accounting for a decrease in 3.5 out of 30 routinely available CSICU nursing positions. Cardiac anesthesiologists and operating room nurses were present in each cardiac procedure that was performed during the study period.

### Resources

Unlike personnel changes, there were no significant lack of resources during the study period. Thanks to a massive national campaign promoting blood donations, there was no scarcity of blood products. Any blood type or quantity of blood products were always available. There was, however, a shortage in the supply of Cefazolin, which is utilized for perioperative wound infection prophylaxis. Cefazolin was replaced by Cefuroxime once needed.

### Statistical analysis

Variable distributions were presented by means and standard deviations (SD) for continuous variables and by frequencies for categorical ones. Discrete variables were calculated using the Fisher test rather than the chi-square test, due to the low incidence of adverse outcomes in the study population. All tests were two-sided. The p-values were calculated using either the Fisher test for discrete variables or the t test for continuous variables and considered significant when less than 0.05 (*p* < 0.05). All calculations were performed with STATA SE software (StataCorp LLC, College Station, TX, USA).

## Results

**Fig. 1:**
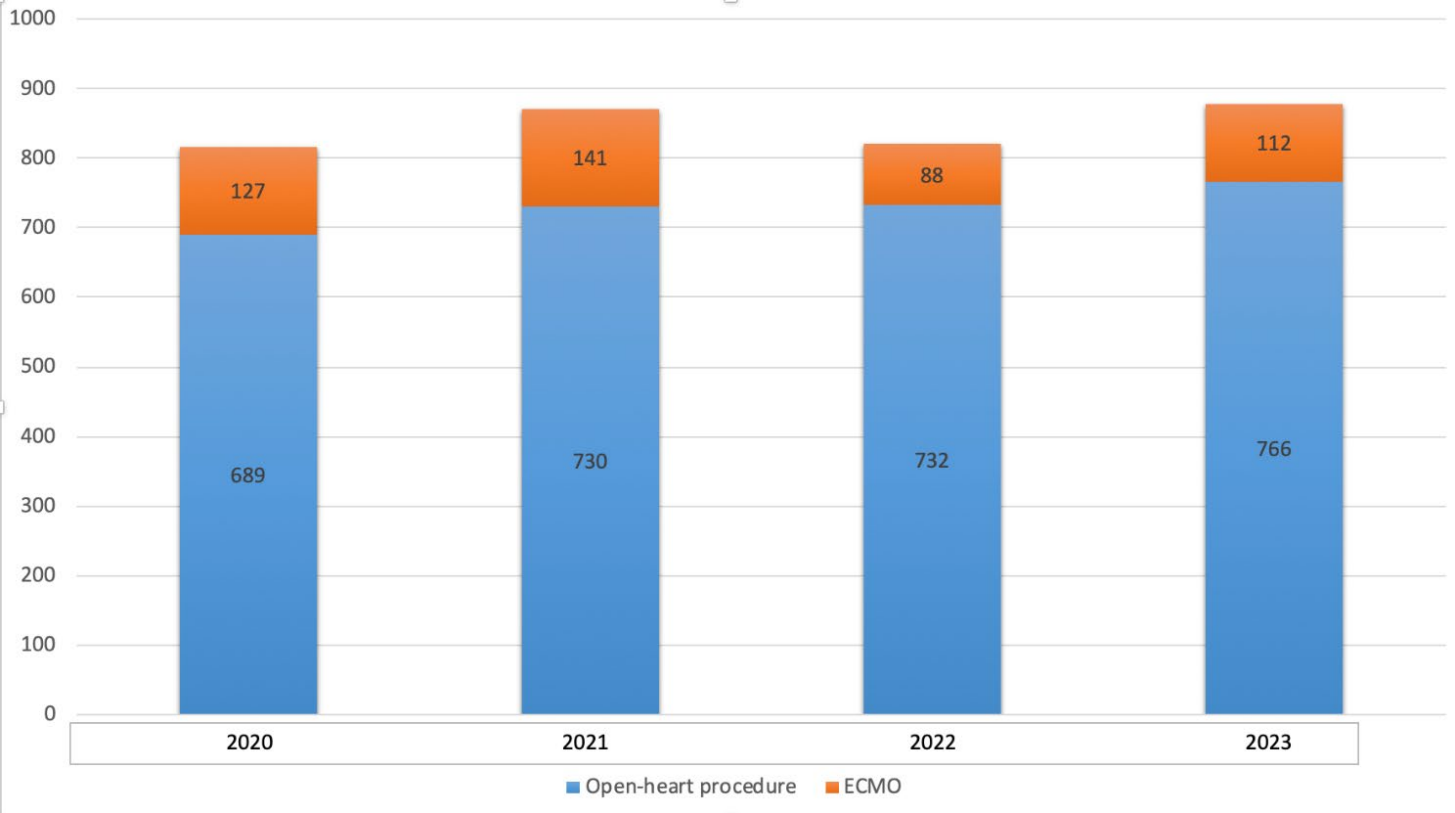
Total cases per year distribution. The yearly total cases that were carried out at the cardiac surgery department at Sheba Medical Center. The Blue section of the bars represent the open-heart number and the Orange sections represents ECMO cannulation procedures. Apart 2020, in which the global pandemic burst in the world, the number of open-heart cases is relatively steady throughout the recent years, with a slight increase in 2023

In the years 2021 and 2022 our department had performed 730 and 732 adult open-heart procedures, respectively. In 2020, amidst the COVID-19 pandemic, only 689 open heart procedures were performed at our institute. In 2023, 766 open-heart procedures have been performed. Approximately 100 cases of ECMO are performed each year at our institute in the last three years, while in 2021 there were 141 cases, also accounting for COVID-19 related ECMO procedures (Fig. 1).

A total of 275 patients were included in the study. 120 patients (43.6%) were operated in 2022 (2022 group) and 155 (56.4%) during wartime in 2023 (2023 group). This signifies a 29.1% increase in the total cases (*p* = 0.26). There were 11 cases (9.1%) of ECMO in 2022 (of which 9 cases of VA ECMO and 2 cases of VV ECMO) and 10 cases (6.4%) in 2023 (of which 8 cases of VA ECMO and 2 cases of VV ECMO). Thus, 109 open-heart cases in 2022 compared to 145 in 2023 account for a 33.0% increase in the number of open-heart operations. However, this increase was not statistically significant (*p* = 0.34).

**Fig. 2:**
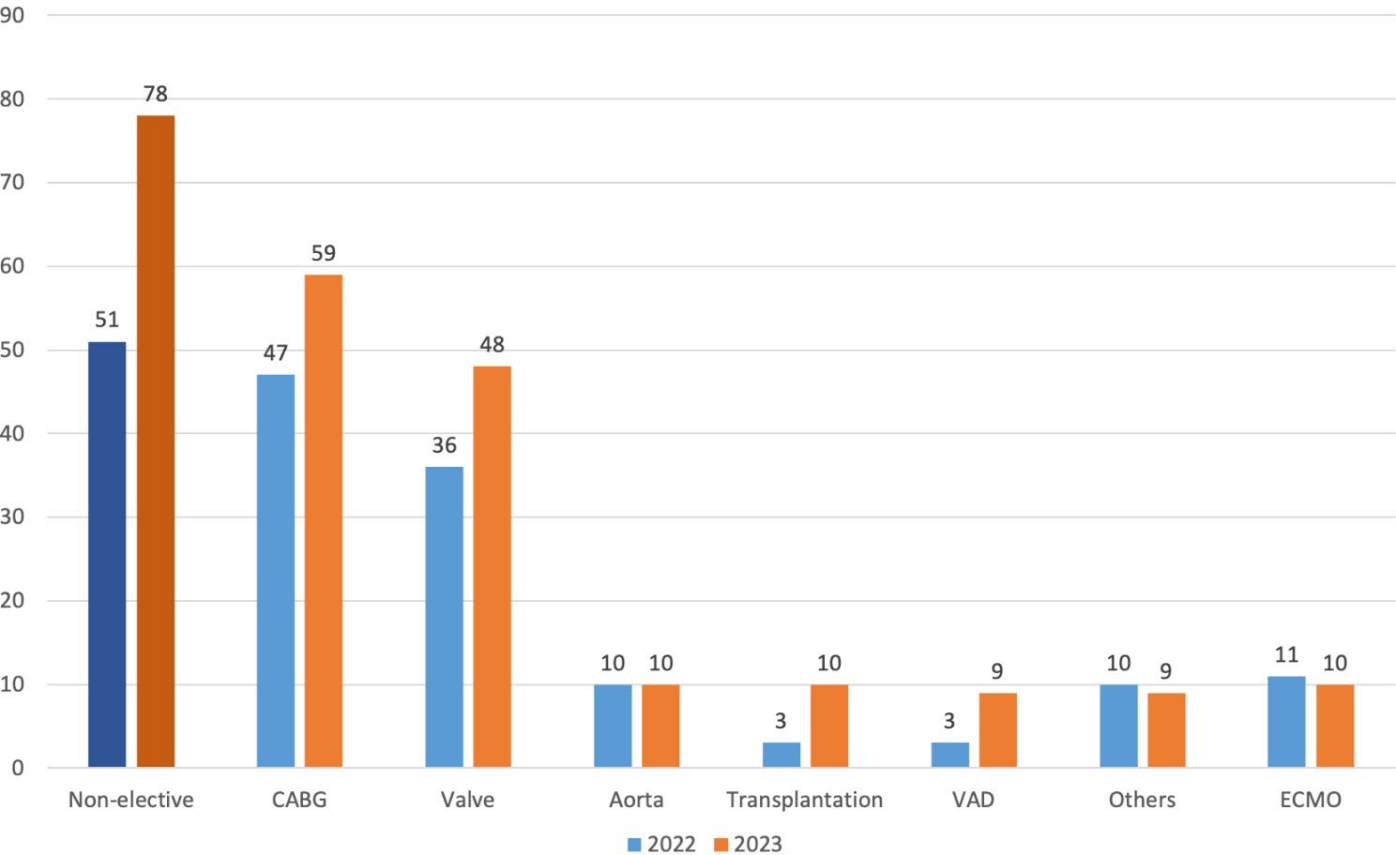
Distribution of procedure type. The chart depicts the distribution of the procedure types between 2022 (Blue) and 2023 (Orange). The first bars represent the non-elective procedures, including emergent and urgent operations. A notable increase in the number of transplantations and VAD implantations. Though higher, none of the procedure types in 2023 were significantly increased from 2023 (p-value non-significant). Other surgery included pulmonary embolectomy, pulmonary endarterectomy, adult congenital cases, on-pump pericardiectomy, septal myectomy and war-related surgeries. *CABG* = Coronary Artery Bypass Grafting; *ECMO* = Extracorporeal Membrane Oxygenation; *VAD* = Ventricular Assist Device

There were no differences in baseline characteristics of the patients in October-November of the last two years. These characteristics included a NYHA class III or IV at presentation as well as mean ejection fraction. There were no significant differences in intraoperative characteristics, which included procedural times and type of surgery, as well as the number of non-elective procedures (Table 1). However, compared to 2022, there was a 233% increase in the number of transplantations (10 in 2023 vs. 3 in 2022, *p* = 0.24), including heart and lungs transplantation. The lack of statistical significance could be explained by the relatively low number of transplants. The number of other cardiac operations performed during wartime was similar to the amount done in previous years. These included coronary artery bypass grafting (CABG) or CABG plus valve surgery (38.0% vs. 39.1% in 2022, *p* = 0.36), isolate valve surgery (30.9% vs. 30.0% in 2022, *p* = 0.38), aorta surgery (6.4% vs. 8.3% in 2022, *p* = 1.00) and other procedures (4.5% vs. 8.3% in 2022, *p* = 0.56), which included pulmonary embolectomy, pulmonary endarterectomy, adult congenital cases, on-pump pericardiectomy, and septal myectomy (Fig. 2). Three cases of war-related trauma were operated (central picture).

**Table 1:** Baseline characteristics. The baseline characteristics and comorbidities of all the patients in the cohort. The groups are comparable in terms of their preoperative conditions

Pre-operative patient characteristics	2022 (*N* = 120, 43.7%)	2023 (*N* = 155, 56.3%)	*p*-value
Age (*years*)	62.48 (± 12.61)	65.09 (± 13.37)	0.10
Gender (*female*)	35 (29.1)	41 (26.5)	0.67
Height (*cm*)	170.38 (± 10.37)	169.80 (± 9.04)	0.63
Weight (*kg*)	79.62 (± 19.46)	78.65 (± 13.71)	0.64
BMI (*kg•m*^*− 2*^)	27.61 (± 8.71)	27.28 (± 4.29)	0.69
**Comorbidities**			
Hypertension	71 (60.7%)	77 ( 59.2%)	0.91
PVD	6 ( 5.6%)	8 (6.8%)	0.90
Diabetes	40 (33.1%)	47 (36.7%)	0.63
Hyperlipidemia	76 (63.3%)	84 (65.6%)	0.80
Renal impairment	12 (10.2%)	11 (8.5%)	0.82
Dialysis	5 ( 4.2%)	1 (0.8%)	0.17
Active smoking	29 (31.5%)	23 (23.5%)	0.28
COPD	9 (8.3%)	9 (7.6%)	1.00
Prior CVA/TIA	4 (3.4%)	10 (7.9%)	0.21
Neurological deficiency	6 (5.0%)	2 (1.6%)	0.24
Heart failure	25 (21.2%)	24 (18.5%)	0.70
Mean LVEF (%)	51.71% (± 14.41)	51.45% (± 15.68)	0.90
Severe pulmonary hypertension	2 ( 2.0%)	4 (3.2%)	0.87
NYHA III/IV	25 (20.5%)	28 (20.9%)	0.43
Arriving in shock state	16 (13.2%)	14 (10.7%)	0.67

*BMI* = Body Mass Index; *COPD* = Chronic Obstructive Lung Disease; *CVA* = Cerebrovascular Accident; *LVEF* = Left Ventricular Ejection Fraction; *NYHA* = New-York Heart Association; *PVD* = Peripheral Vascular Disease; *TIA* = Transient Ischemic Attack. Data is presented as mean ± standard deviation for continuous variable and by frequencies for categorical ones

Regarding postoperative course and outcomes, patients undergoing cardiac surgery during wartime had comparable rates of postoperative complications, including revision for bleeding or tamponade, neurological impairment, postoperative myocardial ischemia (MI), and sternal wound infection. There was a tendency towards a lower rate of tracheostomy (3.5% vs. 8.3% in 2022, *p* = 0.08) and a higher rate of post-pericardiotomy syndrome (PPS) (32.4% vs. 21.5% in 2022, *p* = 0.06). Although not significant, hospital mortality in 2023 was approximately half of the previous year with a rate of 4.5% (*n* = 7) vs. 10.0% (*n* = 12) in 2022 *(p* = 0.1). In 2022, 7 of the 12 (58.3%) mortality cases were ECMO patients and 5 (41.7%) were open heart cases, while in 2023 2 of the 7 (28.5%) mortalities were ECMO patients and 5 (71.5%) were open heart cases. There were relatively high rates of revision in both years (9.1% in 2022 and 7.7% in 2023). The duration of ventilation, the stay in the cardiac surgery intensive care unit (CSICU) and the hospitalization time were comparable in both years, as presented in Table 2.

**Table 2:** Operative data and Postoperative outcomes. The table describes the operative and postoperative short-term outcomes of patients in both groups. No statistically significant differences were shown for any of the parameters that were checked, including in-hospital mortality. Mortality is presented twice: once as total cases of mortality accounting for open-heart mortality and ECMO mortality, and once for open-heart mortality alone

	2022 (*N* = 120, 43.7%)	2023 (*N* = 155, 56.3%)	*p*-value
**Operative data**			
Non-elective surgery	51 (42.5%)	78 (50.4%)	0.65
Bypass time (*minutes*)	123.90 (± 125.54)	108.86 (± 76.06)	0.25
Cross clamp time (*minutes*)	74.79 (± 83.17)	72.66 (± 130.28)	0.88
**Outcomes**			
Revision for bleeding/tamponade	11 (9.1%)	12 (7.7%)	1.00
Stroke	7 (5.8%)	3 (2.1%)	0.21
Gastrointestinal bleeding	1 (0.8%)	0 (0%)	0.93
Postoperative MI	2 (1.7%)	0 (0%)	0.41
Atrial fibrillation	27 (22.3%)	44 (31.0%)	0.15
PPS	26 (21.5%)	46 (32.4%)	0.06
CAVB	2 (1.7%)	5 (3.5%)	0.58
Tracheostomy	10 (8.3%)	5 (3.5%)	0.08
Sternal wound infection	3 (2.5%)	5 (3.5%)	0.88
Ventilation time, *h* (median, IQR)	12.0 (10)	11.0 (9.0)	0.23
CSICU time, *h* (median, IQR)	29.0 (20.00)	40.0 (21.0)	0.36
Hospitalization time, *days* (median, IQR)	7.0 (5.0)	8.0 (6.0)	0.18
Total in-hospital Mortality	12 (10.0%)	7 (4.5%)	0.10
Open-heart in-hospital Mortality	5 (4.5%)	5 (3.4%)	0.10

*CAVB* = Complete Atrioventricular Block; *CSICU* = Cardiac Surgical Intensive Care Unit; *MI* = Myocardial Ischemia; *PPS* = Post-Pericardiectomy Syndrome. Data is presented as mean ± standard deviation for continuous variable and by frequencies for categorical ones

## Discussion

While writing these lines, the war of October 7th, 2023, is still going on. War is an undesired state that impacts every system of a nation, in particular the healthcare system. With the ongoing war in Ukraine, few testimonies have reported that in many centers in Ukraine only urgent cardiac surgeries were performed at the beginning of the war [1, 2]. As the war began in Israel there was massive military recruitment of healthcare professionals, including surgeons. The cardiac surgery department at our institute is currently working with approximately half of the surgeons who were available prior to the attack on October 7th. In addition, a number of nurses were recruited for army service. Initially, we expected a decrease in the number of open-heart procedures, such as the one that occurred in Ukraine and at the beginning of the 2020 pandemic [1]. Nevertheless, 145 open-heart cases were performed in 2023 during the first two months of the war at our center compared to 127 and 109 open-heart cases that were carried out from October 7th – December 7th in 2021 and 2022, respectively. This accounts for a 14% increase from 2021, and a 33% increase from 2022. It should be noted that the other cardiac surgery centers in the region were still operating during the study period. In this unprecedented situation, we sought to evaluate this phenomenon and characterize the patients who were treated during this time of war. One hypothesis previously described mentions that the stressful state of war might trigger or worsen ischemic heart disease [3]. Therefore, when conducting this study, we expected to see patients present in a higher NYHA class along with a surge in CABG procedures. However, analyzing the results of this study showed that the characteristics of the patients during October-December 2023 did not differ from those of October-December 2022. Furthermore, the relative number of CABG procedures in 2023 was not significantly higher when compared to 2022, nor the rate of isolated valve procedures (Fig. 2).

War, however, has brought an increase in the number of transplantations performed due to the unfortunate increase in the pool of donors, mostly brain wounded soldiers. 10 transplant cases have been performed from October 7th 2023 – December 7th 2023 compared to only three transplant cases in October 7th 2022 – December 7th 2022 corresponding to a 233% increase in transplantations over this two-month period in consequent years. As previously mentioned, the rise in transplantations was accompanied by a 50% decrease in transplant surgeons. This situation compelled other surgeons to overtake some of the transplantation workload, such as performing the recipient resternotomy or organ procurement procedures. However, due to the relatively low number of cases, no statistical significance was proven (*p* = 0.24). As the war continues, the number of transplantations will undoubtedly continue to increase. As of December 16th, 2023, three more cases have been successfully carried out. Therefore, it can be concluded that a wartime increase in the number of possible organ donors can be expected in a working healthcare system.

It is worth noting that there was a relatively higher rate of revision in the study period when compared to our annual revision rate. The revision rate during the study period in 2023 was 7.7% compared to 9.1% in 2022 and our center’s annual rate of revision of 5.5%.

Previously reported revision rates range from 2 to 11% [4, 5]. The combination of the complexity of patient cases (undergoing assist device implantations, Type A dissections and more), the inability to fully wait for antiplatelets cessation, and our approach of performing early revisions account for the increase in revision rate. Additionally, a recently reported large-scale study on post-cardiotomy tracheostomy reported better results for early tracheostomy (< 10 postoperative day), which aligns with our traditional approach for patients who, we believe, will require prolonged mechanical ventilation [6]. This explains the higher rates of tracheostomies performed in the study period (8.3% in 2023 and 3.5% in 2022).

**Fig. 3:**
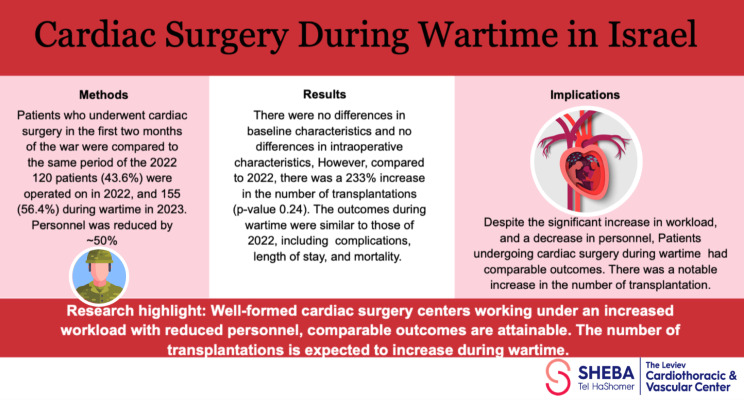
Graphical abstract. The graphical abstract outlines the study’s methods, results and main implication

Lastly, it might be presumed that given the aforementioned challenging circumstances during wartime, the outcomes would be negatively affected. However, the results of this study display that patient outcomes during the wartime period were similar to those of the corresponding period in the previous year. We believe that the competence and flexibility of the workforce underpin the success of the team in producing good results in these highly challenging circumstances. The resilience of cardiac surgery teams amidst difficult situations has previously been described during the COVID-19 pandemic [7]. Unlike that period, experienced by much of the western world during the pandemic, the crisis we face poses an additional issue: the absence of many healthcare providers, including highly skilled surgeons [8, 9]. It is difficult to assess whether the increase in the number of cases during our wartime is incidental or has been indirectly affected by the influence of the war on the behavior of the population. Given the scarcity of reports from Ukraine concerning cardiac surgery during wartime and the fact that no major war has taken place in the western world since the fifties, it is difficult to fully comprehend this phenomenon. However, regardless for the reasons that lead to an increase in the number of cardiac operations, it can be safely concluded that in a well-established and experienced cardiac surgery department, the quality and completeness of care is not worsened but rather improved. It is important to note that no active steps have been taken in real-time to deal with the increase in workload. Surgeons, residents and nursing staff alike, have taken over more tasks than usual and worked longer shifts. Despite risking team burnout in case these circumstances were to continue for a prolonged period of time, no signs of burnout were witness during the study period. On the contrary, there was a strong sense amongst the team members that they were fulfilling a part of the war-efforts. Nonetheless, long-term results data should be collected in the future to better assess these factors (Fig. 3).

## Limitations

This study presents several limitations. First, this is a retrospective study subjected to selection bias in its nature. Second, the lack of similar data and reports available makes it difficult to support the results of this study. Third, the relatively low number of several procedures, such as transplantations, ECMO cases, and miscellaneous operations, could have affected the power of their statistical analysis. Fourth, this study lacks information regarding how operational were the other cardiac surgery departments in the region. It is known that these departments had been operating during the study period, however data regarding the specific number of operations is not available. This data may help explain the increase in the number of operations during the wartime. Finally, given the study design, long-term results were not attainable.

## Conclusion

This study was designed to characterize and evaluate the performance of the largest cardiac surgery center in Israel during the current war. We have experienced an increase in our workload accompanied by a decrease in available personnel. The results of this study demonstrated that patients who underwent cardiac surgery during wartime presented similarly to those of the same period last year and had comparable outcomes. Although there were no statistically significant differences in the rates of the various procedures, there was a trend in the number of transplantations this year.

## Data Availability

Supporting data are available upon reasonable request.

## Abbreviations

BMI: Body Mass Index
CABG: Coronary Artery Bypass Grafting
CAVB: Complete Atrioventricular Block
COPD: Chronic Obstructive Lung Disease
CSICU: Cardiac Surgical Intensive Care Unit
CVA: Cerebrovascular Accident
ECMO: Extracorporeal Membrane Oxygenation
IDF: Israeli Defense Forces
LVEF: Left Ventricular Ejection Fraction
MI: Myocardial Ischemia
NYHA: New-York Heart Association
PPS: Post-Pericardiectomy Syndrome
PVD: Peripheral Vascular Disease
TIA: Transient Ischemic Attack

## Declarations

### Central message

Well-formed cardiac surgery centers under an increased workload with absent personnel, comparable outcomes are attainable. The number of transplantations is expected to increase during wartime.

### Perspective statement

The largest cardiac surgery center in Israel has seen significant increases in workload during the ongoing war in the region despite many surgeons serving in military service. This study characterizes the outcomes of the patients undergoing cardiac surgery during wartime, and provides insights on the importance of a well-formed cardiac surgery team operating during a national crisis.
